# Degradation of MUC7 and MUC5B in Human Saliva

**DOI:** 10.1371/journal.pone.0069059

**Published:** 2013-07-18

**Authors:** Sachiko Takehara, Masaki Yanagishita, Katarzyna Anna Podyma-Inoue, Yoko Kawaguchi

**Affiliations:** 1 Section of Oral Health Promotion, Department of International Health Development, Division of Public Health, Graduate School of Dental and Medical Sciences, Tokyo Medical and Dental University, Tokyo, Japan; 2 Section of Biochemistry, Department of Hard Tissue Engineering, Division of Bio-Matrix, Graduate School of Dental and Medical Sciences, Tokyo Medical and Dental University, Tokyo, Japan; National Institute of Agronomic Research, France

## Abstract

**Background:**

Two types of mucins, MUC7 and MUC5B constitute the major salivary glycoproteins, however their metabolic turnover has not been elucidated in detail to date. This study was conducted to examine turnover of MUC7 and MUC5B in saliva, by focusing on the relationship between their deglycosylation and proteolysis.

**Methodology/Principal Findings:**

Whole saliva samples were collected from healthy individuals and incubated at 37°C in the presence of various protease inhibitors, sialidase, or a sialidase inhibitor. General degradation patterns of salivary proteins and glycoproteins were examined by SDS-polyacrylamide-gel-electrophoresis. Furthermore, changes of molecular sizes of MUC7 and MUC5B were examined by Western blot analysis. A protein band was identified as MUC7 by Western blot analysis using an antibody recognizing an N-terminal epitope. The MUC7 signal disappeared rapidly after 20-minutes of incubation. In contrast, the band of MUC7 stained for its carbohydrate components remained visible near its original position for a longer time indicating that the rapid loss of Western blot signal was due to the specific removal of the N-termimal epitope. Pretreatment of saliva with sialidase facilitated MUC7 protein degradation when compared with samples without treatment. Furthermore, addition of sialidase inhibitor to saliva prevented proteolysis of N-terminus of MUC7, suggesting that the desialylation is a prerequisite for the degradation of the N-terminal region of MUC7. The protein band corresponding to MUC5B detected in both Western blotting and glycoprotein staining showed little sign of significant degradation upon incubation in saliva up to 9 hours.

**Conclusions/Significance:**

MUC7 was highly susceptible to specific proteolysis in saliva, though major part of MUC5B was more resistant to degradation. The N-terminal region of MUC7, particularly sensitive to proteolytic degradation, has also been proposed to have distinct biological function such as antibacterial activities. Quick removal of this region may have biologically important implication.

## Introduction

**Figure 1:**
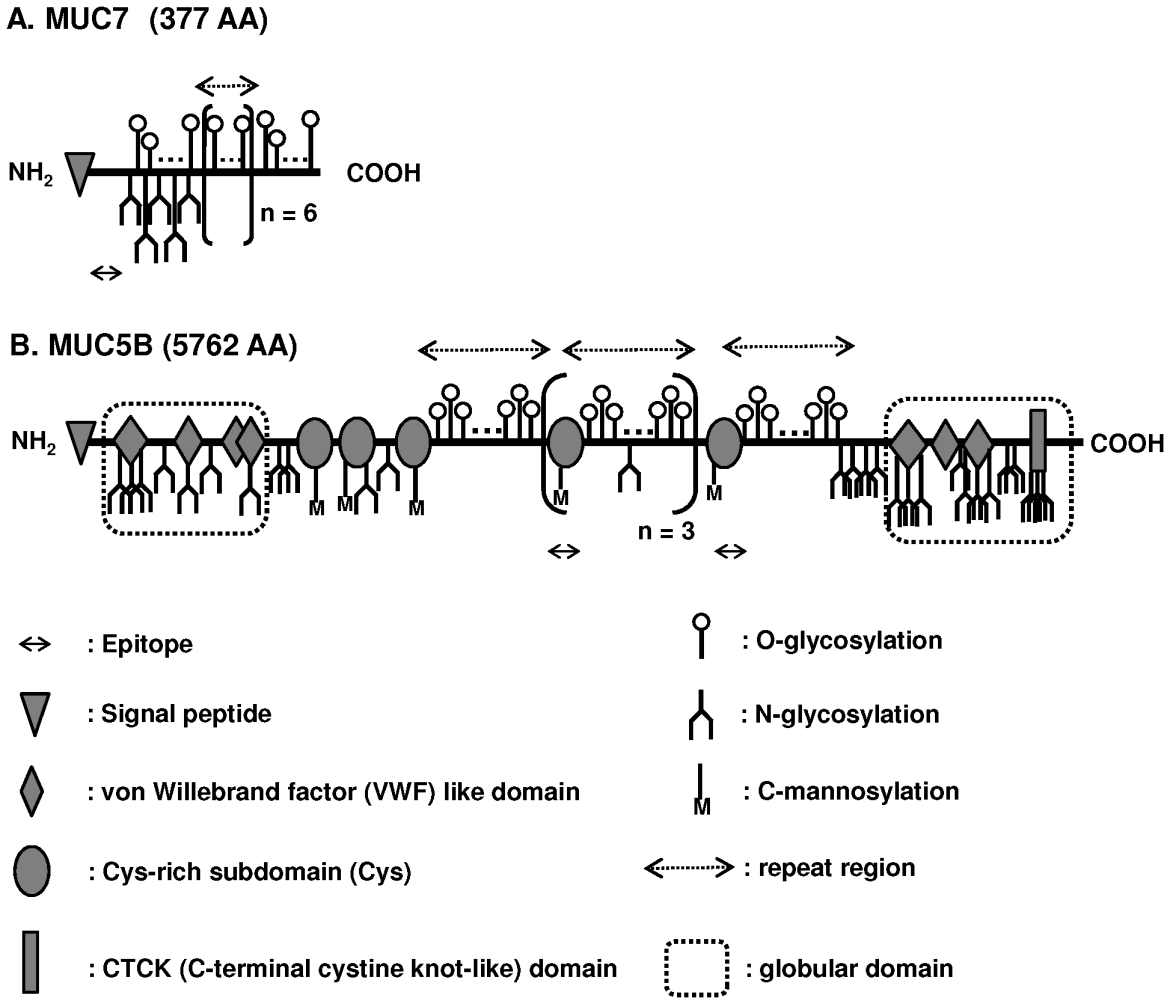
Schematic structures of MUC7 (A) and MUC5B (B), and epitopes recognized by anti-MUC7 and anti-MUC5B antibodies. MUC7 and MUC5B are heavily glycosylated by oligosaccharide side chains. Positions of putative N- and O-glycosylation sites, and C-mannosylation sites are marked. O-linked sugars possess terminally located sialic acid residues that were omitted for simplicity. EU-MUC7a, a mouse monoclonal antibody recognizes the amino acid sequence EGRERDHELRHRRHHHQ, located in the N-terminal domain (amino acids 21–37) of MUC7. EU-MUC5Bb (IgG1 subclass) is a mouse monoclonal antibody which recognizes amino acid sequence RNREQVGKFKMC, located in four of the cysteine-rich domains of the tandem repeat of MUC5B (amino acids 2388–2399, 2917–2928, 3615–3626, 4144–4155). The figures were drawn based on the information in Uniprot (accession number Q9HC84 and Q8TAX7).

Human whole saliva is a solution comprising exocrine secretions from the major and minor salivary glands mixed with the nonexocrine constituents including gingival crevicular fluid, oral epithelial cells, bacteria, and their metabolic products. Saliva contains a complex mixture of proteins with different biological roles in digestion, lubrication, and host defense [1]. Salivary mucins are one of the major components of saliva, comprising nearly 20% of the total salivary proteins [1]. Mucins are high-molecular weight glycoproteins secreted from sublingual, submandibular and minor salivary glands. MUC7 and MUC5B are the two major mucins in saliva (Fig. 1). MUC7 is known as a low-molecular weight, monomeric mucin with the molecular mass of approximately 130–180 kDa [1]. MUC5B is known as a high-molecular weight, oligomeric mucin with the total molecular mass of 2–4×10^4 ^kDa [1]. Both mucins are highly C, N and O-glycosylated, and 40–80% of their sugar chains are O-linked oligosaccharides capped with sialic acids [2]–[4]. The mucins in human saliva are potent lubricants and provide an effective barrier against desiccation [2]. They can also form molecular complexes with other salivary proteins [5]. Many of such protein complexes bind to bacteria and cause their agglutination, facilitating their clearance from the oral cavity [5]. For example, MUC7 contains a histatin-like binding domain against bacteria at its non-glycosylated N-terminus [6], which is known to work as an antifungal and antibacterial peptide [7].

Both MUC7 and MUC5B have a large central peptide domain with repeated amino acid motifs enriched in serine and/or threonine residues, which are the sites for extensive modifications with O-glycans [8]. In glycoproteins like mucins, their carbohydrate components have been shown to endow the molecules with anti-proteolytic properties [9]. Therefore, removal of carbohydrate chains could make mucins more susceptible to proteolytic degradation [9]. Carbohydrate components can also serve as binding sites for various pathogens and toxins [10]. Sialic acids can sometimes provide charge repulsion, avoiding unfavorable interactions [10]. Presence of sialic acid can also modulate metabolic clearance of some proteins, especially under pathological conditions such as infections by sialidase-producing bacteria [10]. Some of both pathogenic and non-pathogenic bacteria can utilize sialic acids as a nutrient source after releasing them by sialidases. The levels of sialic acids in serum are often significantly elevated under pathological conditions [11].

In deglycosylation of oral glycoproteins, several bacterial species have been implicated in their concerted actions [12]. It has been reported that oral *Streptococci* express a wide range of glycosidases including sialidases, and also most of oral *Streptococci* can use mucin as a nutrient source [12]–[15]. The presence of other sugar sources such as glucose in saliva is reported to inhibit, not only deglycosylation but also proteolysis of glycoproteins possibly by changing nutritional requirement of bacteria [9]. In addition, relationship between deglycosylation and proteolysis, and the mechanism how deglycosylation could affect proteolysis of glycoproteins are still unclear.

We hypothesized that deglycosylation of salivary proteins precedes proteolysis, and make them more susceptible to proteolysis. Hence, this study was conducted to clarify physiological turnover of the two major salivary mucins, MUC7 and MUC5B in saliva, by elucidating relationship between deglycosylation and proteolysis.

## Results

### Half-lives of MUC7 and MUC5B in Saliva

**Figure 2:**
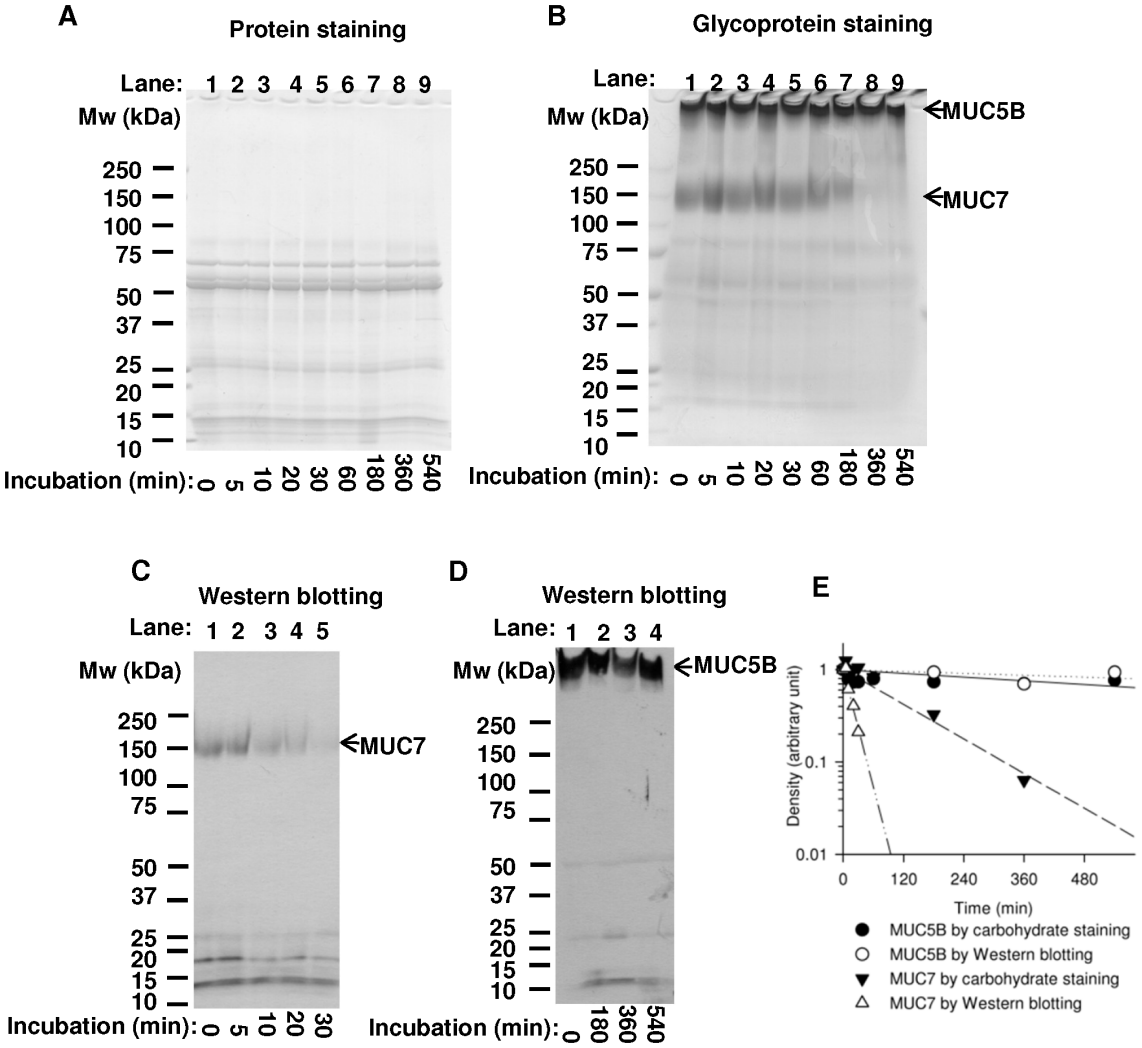
Analysis of salivary protein degradation. Saliva was incubated for up to 9 hours at 37°C, followed by SDS-PAGE. Resolved samples were stained for proteins (A), glycoproteins (B), or incubated with anti-MUC7 (C) and anti-MUC5B (D) antibodies. Arrows in figure C and D indicate the positions of MUC7 and MUC5B. Migration positions of molecular weight standard are shown on the left. In figure (E), the results of glycoprotein staining and Western blotting were analyzed densitometrically, and plotted in a semi-log scale. The linear regression curves and half-life times were then calculated. (•): MUC5B (estimated by glycoprotein staining); (○): MUC5B (estimated by Western blotting); (▾): MUC7 (estimated by glycoprotein staining); (Δ): MUC7 (estimated by Western blotting). Results of one representative sample are shown.

Degradation of salivary proteins in saliva during incubation at 37°C was studied by SDS-PAGE. After the SDS-PAGE, samples were analyzed by protein staining (Fig. 2A), carbohydrate staining (Fig. 2B), or Western blotting (Fig. 2C and D), revealing proteolytic degradation of salivary proteins after incubation. In protein staining, as many as 10 distinct bands were observed (Fig. 2A), and no clear change in density could be seen in these bands after incubation up to 9 hours (Fig. 2A, lane 9). In carbohydrate staining, 2 major and several minor bands were observed (Fig. 2B). Two broad bands with the apparent molecular weight more than 150 kDa or 250 kDa corresponded to MUC7 or MUC5B as identified by the Western blot analysis using antibodies against MUC7 and MUC5B (Fig. 2C and D). In protein staining, no band at the position of MUC7 or MUC5B was recognizable (Fig. 2A), because of their poor stainability due to heavy glycosylation.

Intensity of bands corresponding to MUC7 decreased more rapidly. This was accompanied neither by appearance of a new band nor darkening of other existing bands (Fig. 2B and C), indicating that the destruction of N-terminal epitope recognized by anti-MUC7 antibody occurred rapidly without generating intermediates recognizable by the presence of the epitope. Alternatively, small peptides containing the epitope might have been generated, but they were no longer recognizable by the antibody. In Western blot analysis, bands corresponding to MUC7 disappeared after 20 min incubation (Fig. 2C), while those corresponding to MUC7 in carbohydrate staining did so more slowly, and were still evident at 1 hour of incubation (Fig. 2B). In contrast to MUC7, intensity of bands corresponding to MUC5B exhibited only slight decrease after 9 hours incubation (Fig. 2B and D), with little change in their migration position without appearance of new faster migrating bands.

For estimation of half lives of MUC7 and MUC5B, band densities in the results of carbohydrate staining and Western blotting were measured densitometrically. Figure 2E shows disappearance of MUC7 and MUC5B bands detected by carbohydrate staining (filled symbols) and Western blotting (unfilled symbols). Estimated half-life of MUC7 calculated from the result of Western blot analysis was 12.6±1.6 minutes (mean ± SEM, n = 7), while the one estimated by carbohydrate staining was significantly longer; 290±94 minutes (mean ± SEM, n = 7), indicating that N-terminal epitope of MUC7 was degraded faster than the bulk sugar chains of MUC7. Even after loss of MUC7 epitope (Fig. 2D), apparent molecular weight of the remaining MUC7 was almost as large as or even slightly larger than that of the original MUC7 (Fig. 2B). Estimated half-lives of MUC5B of 2 saliva samples based on the result of carbohydrate staining were longer than 24 hours.

**Figure 3:**
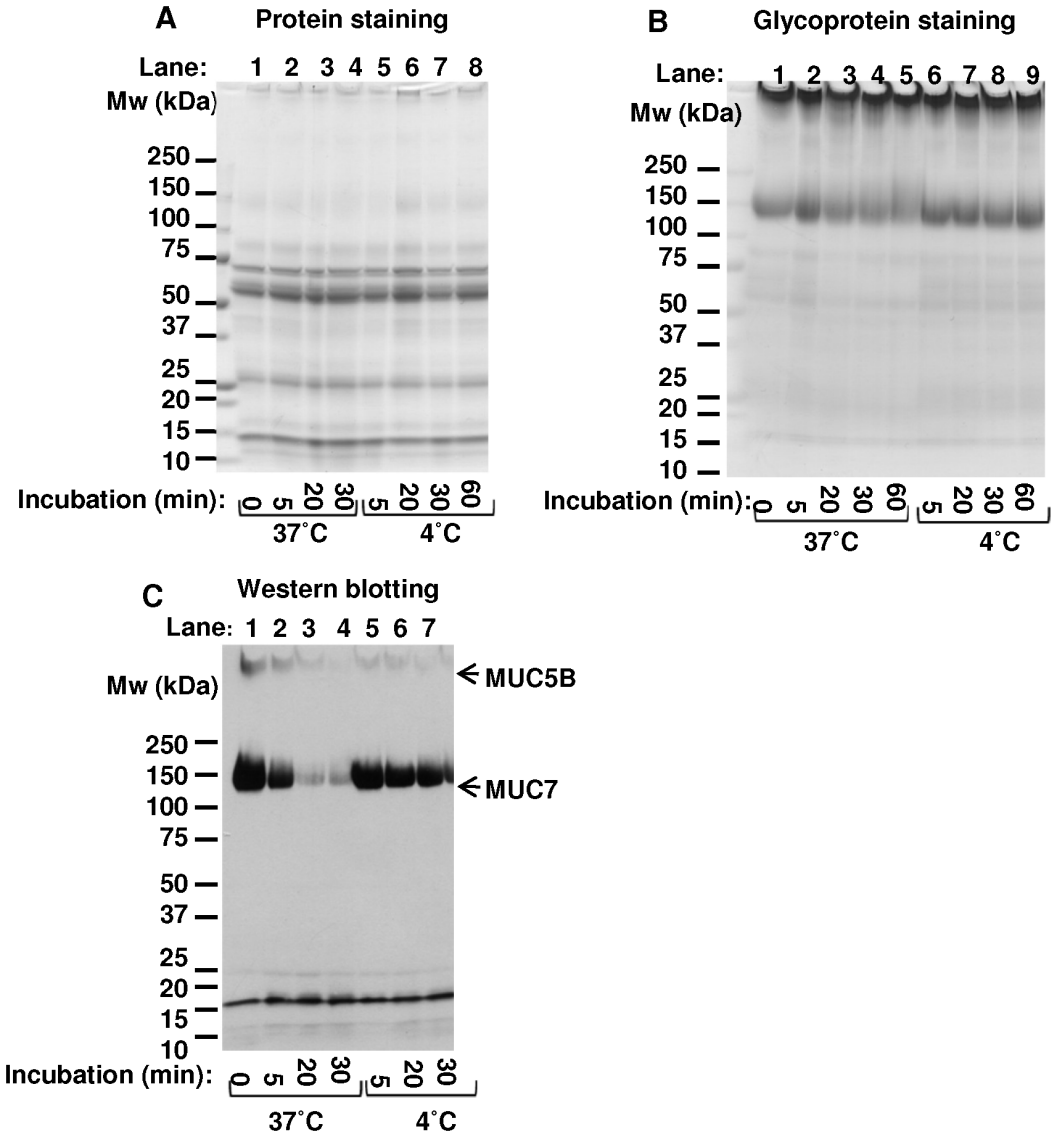
SDS-PAGE analysis of salivary mucins after incubation at 37°C or at 4°C. Incubated saliva samples at 37°C or 4°C were analyzed by SDS-PAGE. Resolved samples were stained for proteins (A), glycoproteins (B), or incubated with anti-MUC7 and anti-MUC5B (C) antibodies. A: Protein staining of salivary proteins after incubation at 37°C for 0, 5, 20 or 30 min (lanes 1–4), or kept on ice for 5, 20, 30 or 60 min (lanes 5–8). B: glycoprotein staining of salivary proteins after incubation at 37°C for 0, 5, 20, 30 or 60 min (lanes 1–5), or keeping on ice for 5, 20, 30 or 60 min (lanes 6–9). C: Western blotting analysis using anti-MUC7 and anti-MUC5B antibodies after incubation of saliva at 37°C for 0, 5, 20 or 30 min (lanes 1–4), or keeping on ice for 5, 20, 30 or 60 min (lanes 5–8). Arrows in Panel C indicate the positions of bands corresponding to MUC7 and MUC5B. Results of representative saliva sample are shown. Migration positions of molecular weight standards are shown on the left.

In order to examine whether the degradation of MUC7 is temperature dependent, saliva samples were incubated at 37°C and 4°C. When the sample was kept at 4°C, the band corresponding to MUC7 showed little change in the density or migration position by carbohydrate staining (Fig. 3B, lane 6–9), or only slight decrease in the density by Western blot analysis (Fig. 3C, lane 5–8), suggesting that epitope of MUC7 and the rest of the molecule were relatively stable at 4°C.

When incubated at 37°C, the bands at the position corresponding to MUC7 (apparent molecular weight 150 kDa) became fainter and showed some broadening toward the direction of slower migration position as the incubation time increased (carbohydrate staining, fig. 3B, lane 1–5). Under the same incubation condition, the bands corresponding to MUC7 decreased faster when detected by Western blotting than those detected by carbohydrate staining but without appreciable change in their migration position (Fig. 3C, lane 1–4). These results suggested that the newly generated, slower migrating species detected in carbohydrate staining, but undetectable in Western blotting, lost the MUC7 epitope. Additional experiments suggested that this slower migration was likely due to the loss of negative charges by desialylation (see below).

### Effects of Sialidase on MUC7

**Figure 4:**
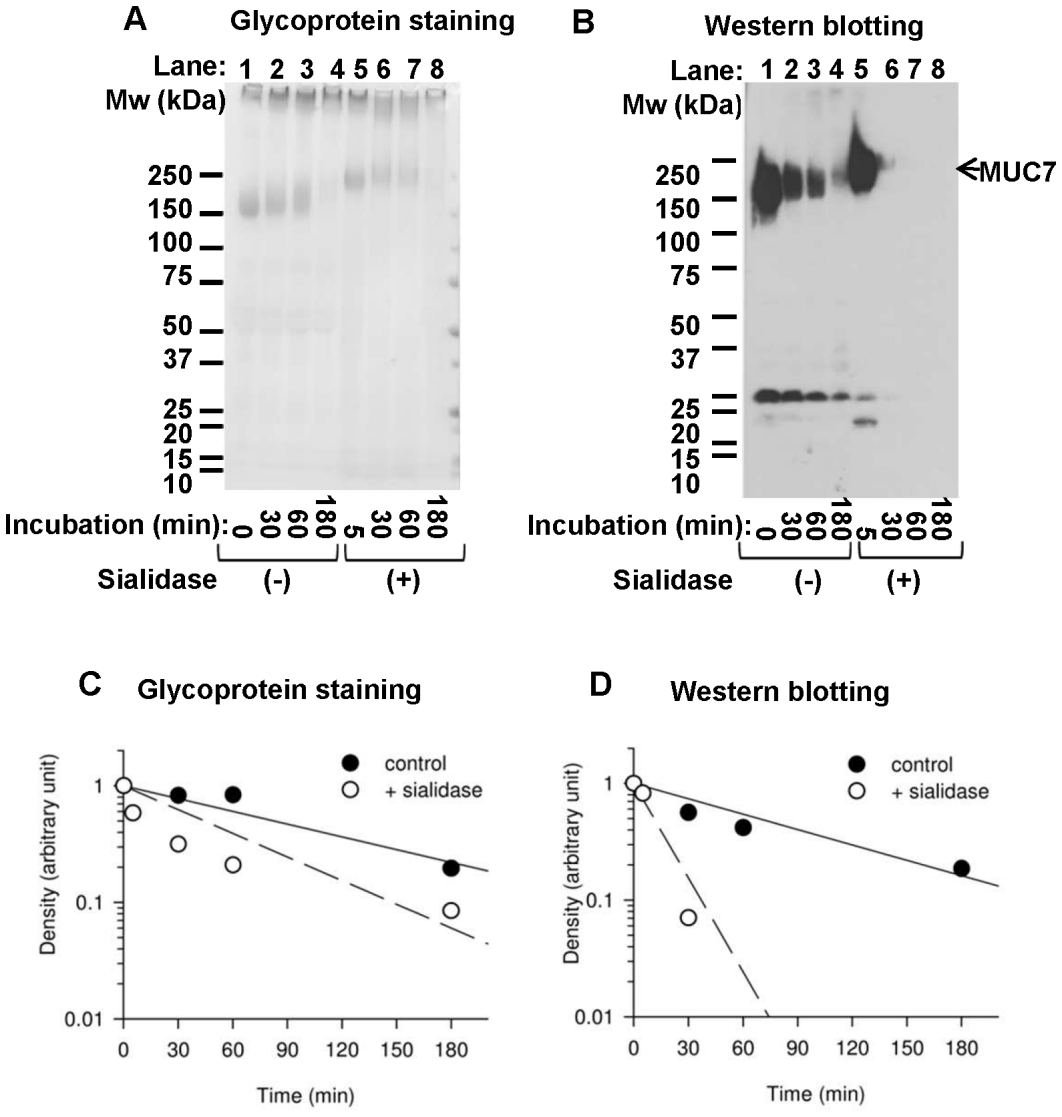
Degradation of MUC7 analyzed by saliva treated with sialidase. Saliva treated with or without sialidase at 37°C up to 3 hours was analyzed by SDS-PAGE. Results of representative saliva sample are shown. Molecular weight standards are indicated on the left of the results. A: glycoprotein staining; B: Western blotting using anti-MUC7 antibody. The arrow in Panel B indicates the position of bands corresponding to MUC7. Saliva was incubated at 37°C for 0, 30, 60 or 180 min without sialidase (lanes 1–4 in A and B) and incubated at 37 °C for 5, 30, 60 or 180 min with sialidase (lanes 5–8 in A and B). Graphs show half-life time of MUC7 estimated by glycoprotein staining (C) and by Western blotting (D). Densities of MUC7 bands were analyzed densitometrically, and plotted in a semi-log scale. Linear regression curves and half-life time were calculated. (•): control (saliva without sialidase); (○): saliva with sialidase.

Effect of desialylation on the electrophoretic mobility of MUC7 was examined. In SDS-PAGE analysis after digestion with sialidase, bands corresponding to MUC7 showed decreased mobility (lane 1 vs. 5 in Fig. 4A and B). Bands detected by Western blotting disappeared rapidly within 30 min of incubation compared with those without enzyme treatment (Fig. 4B). Bands detected by carbohydrate staining were visible even after 60 min incubation. Graphs in figure 4C and D showed effect of sialidase treatment on the degradation of MUC7 in carbohydrate staining and Western blot analysis. Enzymatic removal of sialic acid residues resulted in the slower migration of the MUC7 band, already at 5 min after sialidase treatment (Fig. 4A). Removal of sialic acids, i.e., negative charges, and the preservation of large portion of the MUC7 molecule is responsible for its slower migration.

In this experiment, disappearance of MUC7 after sialidase treatment detected by carbohydrate staining (Fig. 4A) was clearly slower than that detected by Western blotting (Fig. 4B), indicating that the desialylation and the loss of the epitope is sequential. Our results also suggested that the degradation of the epitope preceded that of the rest of the MUC7 molecule as seen in the degradation of intact MUC7 (Fig. 2 and 3).

### Effects of a Sialidase Inhibitor on MUC7

**Figure 5:**
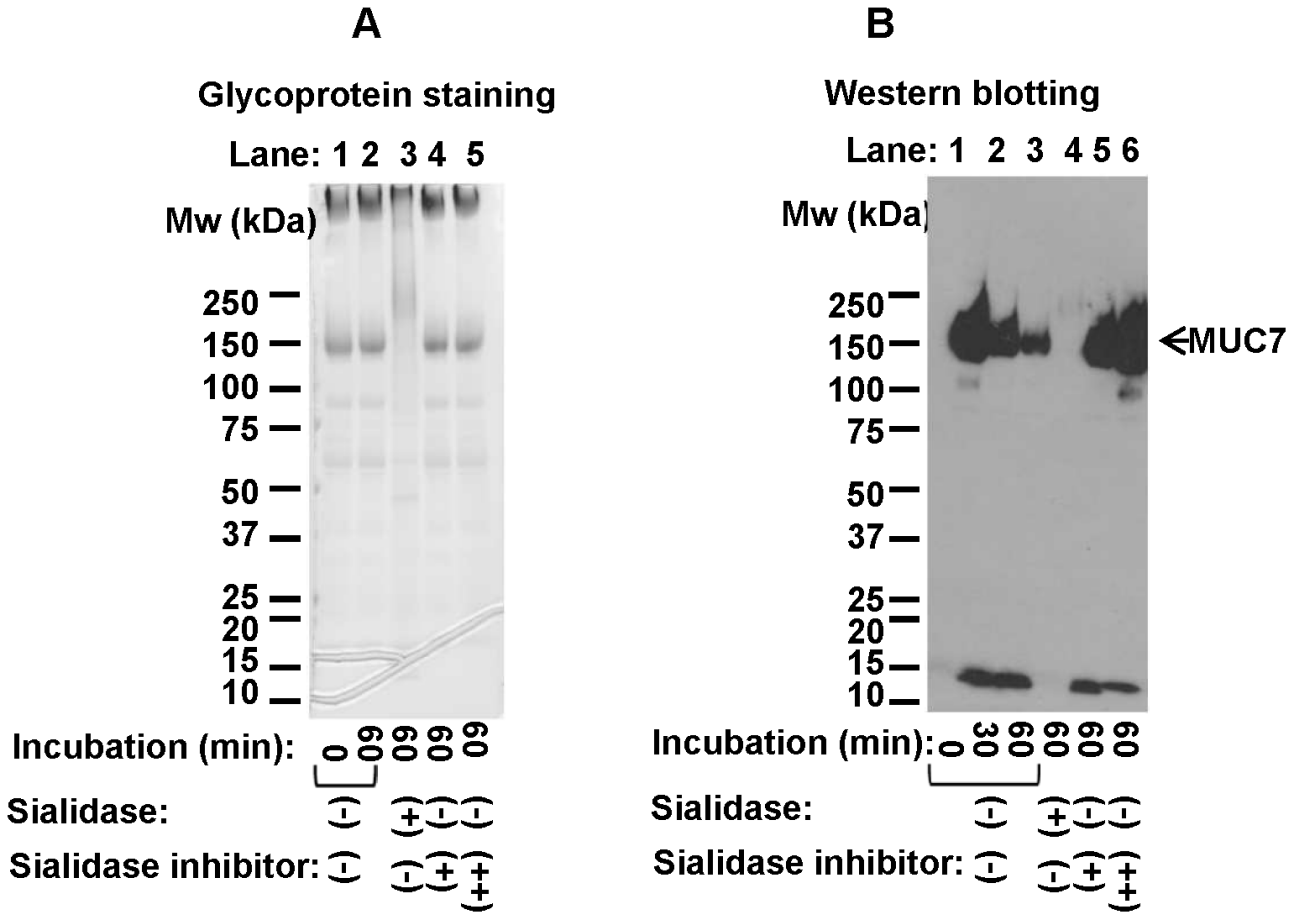
Degradation of MUC7 after treatment of saliva in the presence or absence of sialidase inhibitor. Saliva was incubated with a sialidase or a sialidase inhibitor up to 60 min at 37°C, followed by SDS-PAGE. Results were shown for a representative saliva sample. Migration positions of molecular weight standards are shown on the left. A: glycoprotein staining. B: Western blotting using anti-MUC7 antibody. An arrow in Panel B indicates the position of bands corresponding to MUC7. Saliva was incubated at 37°C as a controlfor 0 and 60 min (lanes 1–2 in A); for 0, 30, and 60 min (lanes 1–3 in B), incubated at 37°C for 60 min with sialidase (lane 3 in A and lane 4 in B); incubated at 37°C for 60 min with 500 µM sialidase inhibitor (lane 4 in A and lane 5 in B); or incubated at 37°C for 60 min with 5 mM sialidase inhibitor (lane 5 in A and lane 6 in B).

In order to clarify the roles of endogenous sialidase, saliva samples were treated with a sialidase inhibitor. After treatment with a sialidase inhibitor, intensity and apparent molecular weight of bands corresponding to MUC7 based on Western blot analysis stayed unaltered after incubation for 60 min (Fig. 5B, lane 1, 5 and 6), indicating that sialidase inhibitor effectively prevented proteolysis of N-terminus epitope of MUC7. Thus, desialylation appears to be a prerequisite for the degradation of the epitope. The observation that the migration position of MUC7 remained unchanged during incubation further supports that the slowed migration of MUC7 seen in Fig. 3B, 4A and B (no sialidase added) was due to desialylation.

### Effects of Protease Inhibitors on Degradation of MUC7

**Figure 6:**
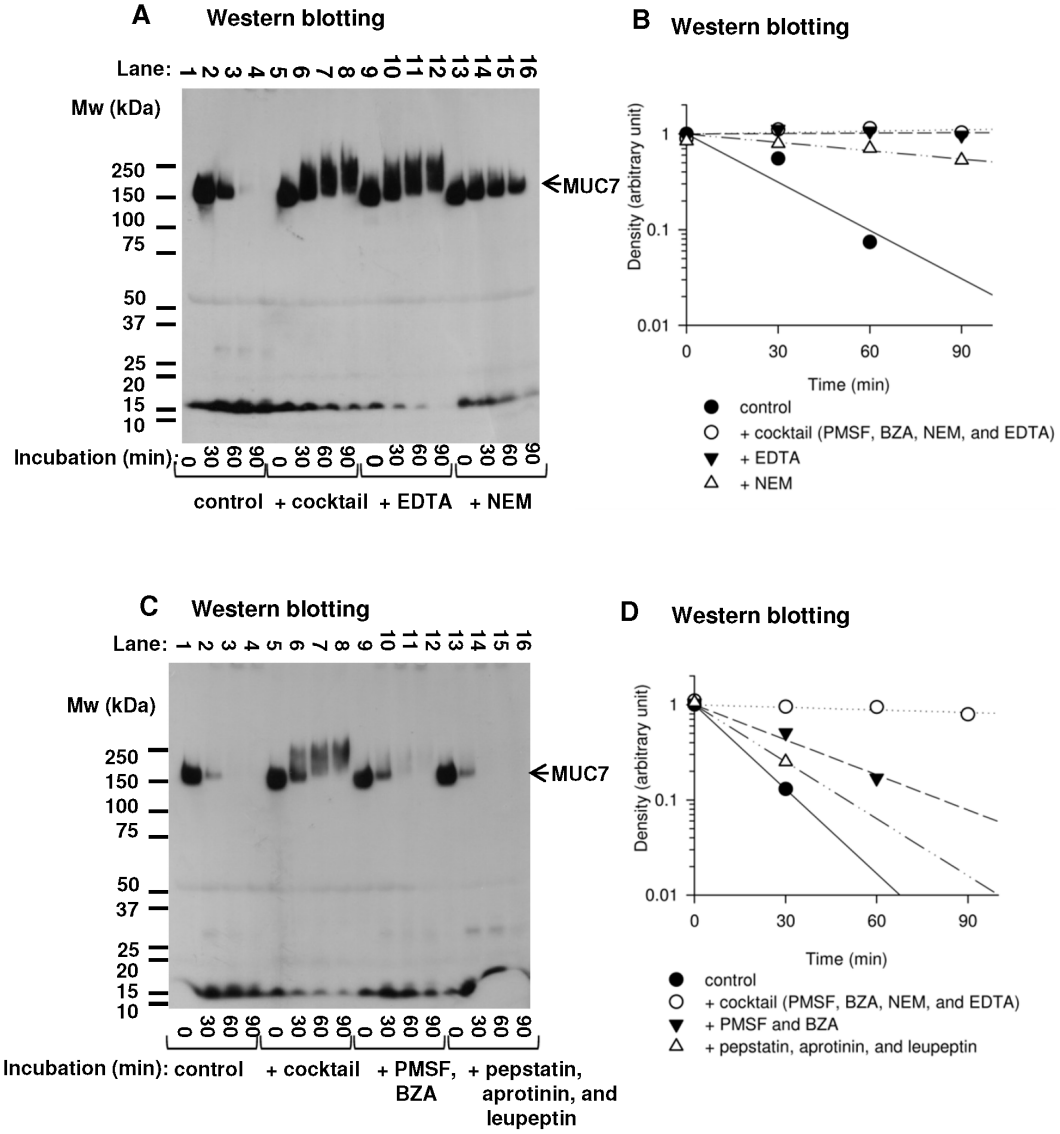
SDS-PAGE analysis of MUC7 after incubation with protease inhibitors. The effects of protease inhibitors on the degradation of MUC7 were determined by Western blotting. Saliva was incubated at 37°C for 0, 30, 60 or 90 min with or without protease inhibitors. Results of a representative saliva sample are shown. Migration positions of molecular weight standards are shown on the left. A: MUC7 was detected by Western blotting. An arrow indicates the position of bands corresponding to MUC7. Lanes 1–4: control (saliva without protease inhibitors); lanes 5–8: saliva with a cocktail of protease inhibitors (i. e., PMSF, BZA, NEM and EDTA); lanes 9–12: saliva with EDTA; lanes 13–16: saliva with NEM. B: A graph shows disappearance of MUC7 in a presence of protease inhibitors. The results of Western blotting were analyzed densitometrically, and plotted in a semi-log scale. Linear regression curves were calculated. (•): control (saliva without protease inhibitors); (○): saliva with a cocktail of protease inhibitors (i.e., PMSF, BZA, NEM and EDTA); (▾): saliva with EDTA; (Δ): saliva with NEM. C: MUC7 was detected by Western blotting. An arrow indicates the position of bands corresponding to MUC7. Lanes 1–4: control (saliva without protease inhibitors); lanes 5–8: saliva with a cocktail of protease inhibitors (i.e., PMSF, BZA, NEM and EDTA) as in lanes 5–8; lanes 9–12: saliva with PMSF and BZA; lanes 13–16: saliva with aprotinin, leupeptin, and pepstatin. D: A graph shows dissapearance of MUC7 in the presence of protease inhibitors. Calculation was done as in figure 2B. (•): control (saliva without protease inhibitors); (○): saliva with a cocktail of protease inhibitors (PMSF, BZA, NEM and EDTA); (▾): saliva with PMSF and BZA; (Δ): saliva with aprotinin, leupeptin, and pepstatin.

Saliva samples were treated with protease inhibitors (Table 1) and incubated at 37°C in order to examine the nature of proteases involved in the degradation of MUC7. Figure 6A and C shows the results of Western blotting of saliva samples. Graphs in figures 6B and D show the disappearance of MUC7 epitope. The cocktail of protease inhibitors or EDTA alone could prevent the degradation of epitope of MUC7 almost completely (Fig. 6A, lanes 5–12). A cysteine protease inhibitor (NEM) was less effective but the bands were still visible after 90 minutes of incubation, suggesting that NEM could partly, but not completely, inhibit the degradation of MUC7 (Fig. 6A, lanes 13–16). The mixture of PMSF and BZA was not effective (Fig. 6C, lane 10–12). The bands of MUC7 in saliva samples incubated with serine protease inhibitors (PMSF and BZA), the other mixture of protease inhibitors (aprotinin, leupeptin, and pepstatin) disappeared as rapidly as those in control (Fig. 6C, lanes 9–16), indicating that those protease inhibitors were ineffective against the degradation of MUC7. By treating saliva sample with the cocktail of protease inhibitors (PMSF, BZA, NEM, and EDTA), generation of slowly migrating species with MUC7 epitope similar to those observed in figure 3B were clearly detectable (Fig. 6A and C, lanes 5–8). These slowly migrating species are likely originated by the loss of negative charges (i.e. desialylation) of MUC7. Also, conversion of MUC7 band into slowly migrating species was clearly observed by preventing proteolytic activity of saliva with a cocktail of protease inhibitors (Fig. 6A and C, lanes 5–8), indicating the presence of sialidase-like activity in saliva.

**Table 1:** Classification of protease inhibitors used.

Classification	Protease inhibitors
Serine proteases	PMSF, BZA, and Aprotinin
Metalloproteases	EDTA
Cysteine proteases	NEM and Leupeptin
Aspartate proteases	Pepstatin
Protease inhibitor cocktail	PMSF, BZA, EDTA, and NEM

## Discussion

In the degradation of glycoproteins, the presence of carbohydrate component often interferes with their proteolysis. We hypothesized that proteolysis of salivary mucins may be preceded by the removal of sugar chains, and enhanced deglycosylation may be accompanied by accelerated proteolytic degradation. Our study demonstrated that MUC7 was degraded at the N-terminal region in a short incubation time at 37°C, and this event was preceded by desialylation. Sialic acids, which cap the non-reducing terminal of sugar chains of MUC7, were suggested to play a key role in modulating the degradation of its non-glycosylated N-terminus peptide domain. Treatment by exogenous sialidase accelerated proteolytic degradation of the non-glycosylated N-terminus of MUC7 and degradation of the remaining part of MUC7 (Fig. 4A and B). In the contrary, a sialidase inhibitor prevented degradation of the N-terminus of MUC7 (Fig. 5B), suggesting that removal of sialic acids from MUC7 likely affected the proteolysis of MUC7. This is consistent with the fact that the presence of sialic acids generally interferes with the proteolytic degradation of glycoproteins [10]. Thus, overall density of sialic acids or one at the specific site of mucins might be a critical determinant of their degradation by limiting access of proteases to protein core. Indeed, MUC7 and MUC5B are considered to be relatively resistant to proteolysis because of their heavy glycosylation. The estimated half-life of N-terminal epitope of MUC7 was short, approximately 10–20 minutes (Fig. 2E), while that of MUC5B was longer than 24 hours (Fig. 2E), though we cannot totally exclude any minor degradation of MUC5B. Estimated half-life of carbohydrate components of MUC7 calculated from carbohydrate staining was longer than that of N-terminal region of MUC7 (Fig. 2E) indicating that, after removal of critical sialic acids, N-terminal region is rapidly degraded, followed by extensive deglycosylation/proteolysis of MUC7 with slower kinetics (Fig. 2B and D). After sialidase treatment, the N-terminus of MUC7 was degraded more rapidly (Fig. 4B), which was in agreement with the above interpretation.

In addition, our experiments provided useful information concerning the handling of saliva samples for protein analyses. For example, our data showed that a sialidase inhibitor could be effective in preventing degradation of MUC7 (Fig. 5A and B). After saliva collection, proper combination of protease inhibitors also can be added to prevent proteolysis. However, inhibition of proteases alone is not sufficient to completely prevent degradation of MUC7, because other enzymes including glycosidases are present in saliva [16] making some salivary proteins such as MUC7 prone to later proteolysis (Fig. 6A, lanes 5–8). With saliva samples kept at 4°C, proteolysis and deglycosylation were both inhibited (Fig. 3B and C), thus keeping collected saliva sample at 4°C in the presence of proper protease and sialidase inhibitors is important. In the present study, the metalloprotease inhibitor (EDTA) and the cysteine protease inhibitor (NEM) most effectively inhibited the proteolysis of MUC7 (Fig. 6), indicating the possibility of major roles of metalloproteases and cysteine proteases at least with those samples examined.

Incubation of MUC7 with sialidases has been reported to decrease antibacterial activity of the protein [17]. Sialidases, mainly produced by *Streptococci* including *S. oralis* are the major mucosal surface infection determinants [18]. Deglycosylation can produce structural alteration in terminal sugar epitopes resulting in generation of new ligands for oral bacteria. Thus, desialylation may affect the affinity of mucins for oral bacteria. The mobility of mucins in SDS-PAGE is significantly influenced by the negative charges of terminal sialic acid residues [16]. In the present study, bands of MUC7 in carbohydrate staining showed the loss in mobility, suggesting decrease of its negative charge with incubation time (Fig. 4A). Desialylation by sialidases is likely to be responsible for this phenomenon.

In conclusion, the present study provides new and important information about degradation of MUC7 and MUC5B in saliva. MUC7 might be degraded from the N-terminal region in short incubation time, this proteolytic degradation seems to be preceded by desialylation. MUC7, after its degradation at N-terminal region, may alter its bioactivity or interactions with other various pathogens and salivary proteins, hence affecting the clearance of salivary proteins and various pathogens. MUC5B is heavily glycosylated and can form multimer by disulfide bonds [19]. Multimerization may account, at least in parts, for its long half-life. Saliva is constantly secreted and swallowed, but the protein may be unevenly cleared depending on its location in oral cavity [20]. This should also be taken into consideration in evaluating metabolic clearance of salivary mucins *in situ*. Further study is needed to elucidate detailed degradation patterns of salivary proteins. A short half-life of MUC7 and a long half-life of MUC5B in saliva were consistently observed among healthy individuals examined in the present study. However, analysis of saliva from additional healthy subjects and of those with diseases would provide further information on the metabolism of salivary proteins.

## Materials and Methods

### Collection of Resting whole Saliva

Resting whole saliva from 7 healthy volunteers (3 females; age 29–50, 4 males; age 24–49) secreted between 9 a.m. and 11 a.m. was collected by spitting into sterile 50 ml tubes, which were maintained on ice during collection. Saliva samples were frozen immediately and stored at −80°C until analysis. All the samples were analyzed simultaneously. The study protocol was approved by the Ethics Committee for Human Research, Tokyo Medical and Dental University (No. 270), and all subjects enrolled in the study signed an informed consent form.

### Antibodies

EU-MUC7a and EU-MUC5Bb (IgG1 subclass) are mouse monoclonal antibodies obtained from the European Consortium (Concerted Action contract number BMH4-CT98-3222). Schematic structures of mucins and positions of epitopes recognized by the antibodies are shown in Figure 1.

### Quantification of Total Salivary Proteins

After thawing at 4°C, saliva samples were clarified by centrifugation at 600 × g for 10 minutes at 4°C. The amount of total proteins in saliva was determined spectrophotometrically at 562 nm with a BCA Protein Assay Kit (Pierce Biotechnology, Inc., Rockford, IL, USA) using bovine serum albumin as a standard.

### Sample Preparation

In order to monitor degradation of salivary proteins, each saliva sample containing 10 µg or 50 µg protein was incubated under one of the four following conditions. The first, saliva was incubated at 37°C. The second, saliva was kept on ice. The third, saliva mixed with cocktails of protease inhibitors (Table 1) was incubated at 37°C. The final concentrations of the protease inhibitors used were: phenylmethyl sulfonyl fluoride (PMSF), 0.1 mM; benzamidine-HCl (BZA), 5 mM; N-ethylmaleimide (NEM), 10 mM; ethylenediaminetetraacetic acid (EDTA), 25 mM; pepstatin, 1.5 µM; leupeptin, 4.7 µM; and aprotinin, 0.15 µM. The forth, saliva mixed with a sialidase or a sialidase inhibitor, was incubated at 37°C. A sialidase originated from *Clostridium perfringens* (EC 3.2.1.18, Roche, Mannheim, Germany) was used at the final concentration of 10 U/ml in 50 mM sodium acetate pH 4.5 buffer. A sialidase inhibitor, N-acetyl-2, 3-didehydro-2-deoxyneuraminic acid (Tokyo Chemical Industry Co., Ltd., Tokyo, Japan) was used at the final concentration of 500 µM or 5 mM. Incubation times are indicated in the text.

### Sodium Dodecyl Sulfate-polyacrylamide Gel Electrophoresis (SDS-PAGE) and Western Blotting

Each saliva sample was dried in a lyophilizer, and reconstituted at two protein concentrations (10 µg/15 µl and 50 µg/10 µl) in sodium dodecyl sulfate (SDS) sample buffer (0.28 M Tris-HCl, pH 6.8, containing 30% (v/v) glycerol, 1% (w/v) SDS, 0.5 M dithiothreitol, and 0.0012% bromphenol blue), incubated at 100°C for 5 min, followed by SDS-PAGE on 4–20% gradient gel (Daiichi Pure Chemicals Co., Ltd., Tokyo, Japan) according to the Laemmli method [21]. Quadruple of samples with protein concentrations at 10 µg/15 µl or 50 µg/10 µl were analyzed by protein staining, carbohydrate staining and Western blotting using anti-MUC5B and anti-MUC7 antibodies. All electrophoretic gels included a common salivary sample as an internal control to validate results. After the electrophoresis, one gel (samples at 10 µg/15 µl protein concentration) was stained for proteins using GelCode Blue (Pierce Biotechnology, Inc., IL, USA) and another one (samples with 50 µg/10 µl protein concentration) for glycosylated proteins by GelCode Glycoprotein Staining Kit (Pierce Biotechnology, Inc., IL, USA). GelCode Glycoprotein Staining Kit detects the presence of carbohydrates based on PAS staining. Salivary protein samples for Western blotting analysis were transferred onto PVDF membranes (Millipore Corporation, Bedford, MA, USA) using wet transfer systems with 15 mV constant voltage for a single chamber for overnight at 4°C. After transfer, membranes were blocked with 1.0% (w/v) bovine serum albumin in phosphate-buffered saline containing 0.1% Tween 20 for 40 min, incubated overnight at 4°C with EU-MUC7a or EU-MUC5Bb antibodies diluted 1∶100 and washed, followed by incubation at room temperature with anti-mouse HRP-conjugated antibody (Pierce Biotechnology, Inc., IL, USA) diluted 1∶1000 for 1 hour. Finally, bands corresponding to MUC7 and MUC5B were detected by ECL Western Blotting Detection System or by ECL Plus Western Blotting Detection System (GE Healthcare Ltd., Buckinghamshire, UK). Stained gels and Western blotting images were scanned with a 8-bit color image scanner (Seiko Epson Corporation, Nagano, Japan), and digitized image analyses were done with the Scion Image Program (Scion Corporation, MD, USA). The program provided the band densities in 256 gray scale values. Half-lives of MUC7 and MUC5B in saliva were calculated from band densities by linear regression analysis using SigmaPlot software ver. 11 (Hulinks Inc., Tokyo, Japan).
